# Multi-Omics Integration in Mice With Parkinson’s Disease and the Intervention Effect of Cyanidin-3-*O*-Glucoside

**DOI:** 10.3389/fnagi.2022.877078

**Published:** 2022-04-29

**Authors:** Wang Wang, Guoxue Zhu, Yuwen Wang, Wei Li, Shilin Yi, Kai Wang, Lu Fan, Juanjuan Tang, Ruini Chen

**Affiliations:** ^1^School of Medicine & Holistic Integrative Medicine, Nanjing University of Chinese Medicine, Nanjing, China; ^2^Nanjing Hospital of Chinese Medicine Affiliated to Nanjing University of Chinese Medicine, Nanjing University of Chinese Medicine, Nanjing, China; ^3^The Sixth Outpatient Department, Jinling Hospital, Nanjing, China; ^4^College of Traditional Chinese Medicine, College of Integrated Chinese and Western Medicine, Nanjing University of Chinese Medicine, Nanjing, China

**Keywords:** Parkinson’s disease, cyanidin-3-*O*-glucoside, metabolomics, microbiome, Pearson’s correlation analysis

## Abstract

**Background:**

Parkinson’s disease (PD) is a multifactorial degenerative disease of the central nervous system, which affects mostly older adults. To date, research has focused on the progression of PD. Simultaneously, it was confirmed that the imbalances in gut microbiota are associated with the onset and progression of PD. Accurate diagnosis and precise treatment of PD are currently deficient due to the absence of effective biomarkers.

**Methods:**

In this study, the pharmacodynamic study of cyanidin-3-*O*-glucoside in PD mice was used. It intends to use the “imbalance” and “balance” of intestinal microecology as the starting point to investigate the “gut-to-brain” hypothesis using metabolomic-combined 16S rRNA gene sequencing methods. Simultaneously, metabolomic analysis was implemented to acquire differential metabolites, and microbiome analysis was performed to analyze the composition and filter the remarkably altered gut microbiota at the phylum/genera level. Afterward, metabolic pathway and functional prediction analysis of the screened differential metabolites and gut microbiota were applied using the MetaboAnalyst database. In addition, Pearson’s correlation analysis was used for the differential metabolites and gut microbiota. We found that cyanidin-3-*O*-glucoside could protect 1-methyl-4-phenyl-1,2,3,6− tetrahydropy ridine (MPTP)-induced PD mice.

**Results:**

Metabolomic analysis showed that MPTP-induced dysbiosis of the gut microbiota significantly altered sixty-seven metabolites. The present studies have also shown that MPTP-induced PD is related to lipid metabolism, amino acid metabolism, and so on. The 16S rRNA sequencing analysis indicated that 5 phyla and 22 genera were significantly altered. Furthermore, the differential gut microbiota was interrelated with amino acid metabolism, and so on. The metabolites and gut microbiota network diagram revealed significant correlations between 11 genera and 8 differential metabolites.

**Conclusion:**

In combination, this study offers potential molecular biomarkers that should be validated for future translation into clinical applications for more accurately diagnosing PD. Simultaneously, the results of this study lay a basis for further study of the association between host metabolisms, gut microbiota, and PD.

## Introduction

Parkinson’s disease (PD) is an adult neurodegenerative disease characterized clinically by tremors, muscle stiffness, and bradykinesia, which leads to a high rate of misdiagnosis in the clinical setting of PD. It affects approximately 1% of the population aged over 60 years, increasing to approximately 4% by the age of 80 years. Furthermore, the clinical symptoms of patients with PD tend to lag behind the pathological changes. At present, the therapeutic strategy for PD is the classical method, dopamine replacement therapy, which can only ameliorate the motor symptoms and is ineffective in slowing, halting, or reversing disease progression. However, long-term therapy could bring out the development of “motor complications,” including wearing-off, motor fluctuations, and L-dopa-induced dyskinesia (Shao and Le, 2019). Although a series of biomarkers stem from clinical, neuroimaging, and genetic studies, the sensitive and specific biomarkers for PD remain deficient (Trifonova et al., 2020). To date, a number of hypotheses have been presented, such as mitochondrial dysfunction, oxidative stress, accumulation of misfolded aggregates, and inflammation (Bhinderwala et al., 2019; Khan et al., 2020). Despite all these years of research, the pathogenesis of PD is still not completely understood (Milutinović et al., 2020). The screening of reliable biomarkers might result in the development of novel drugs that could provide a more accurate diagnosis of PD progression (Lama et al., 2020).

Metabolomics is routinely used as an emerging technique for investigating metabolic profiling through data mining and bioinformatic analysis (Johnson et al., 2016; Laíns et al., 2019). The biomarkers are endogenous, as well as the metabolism of pharmaceuticals and co-metabolism between the host and gut microbiota (Wu et al., 2021). Metabolomics research possesses enormous potential to contact particular physiological or pathological conditions with genetic, environmental, or physiological elements due to the sensibility of metabolites to small variations of endogenous and exogenous (Nicholson et al., 2002).

Neurodegeneration in PD was associated with gastrointestinal (GI) dysregulation, which was proved by a large number of clinical and neuropathological evidence (Abbott et al., 2001). The “gut-to-brain” hypothesis indicates that the accumulation and aggregation of α-synuclein in the gut and the neurodegeneration in the enteric nervous system (ENS) begin up to 20 years before the neurodegeneration in the central nervous system (CNS) (Braak et al., 2006; Hawkes et al., 2010; Cersosimo and Benarroch, 2012). Immunological, neuroendocrine, and direct neurochemical mechanisms are the main mechanisms between the intestine and brain that are modulated by gut microbiota (Kaur et al., 2021). Simultaneously, intestinal inflammation related to dysbiosis may affect the misfolding aggregation of α-synuclein (Resnikoff et al., 2019). So far, some research has examined the metabolic profiling of PD through several biological samples (Okuzumi et al., 2019; Lichtenberg et al., 2021). However, there is a lack of research on metabolomics combined with gut microbiota, which could link the symbiotic microbiota and health easily. A better understanding of the gut microbiota and metabolomic profile could illuminate the association between host metabolisms, gut microbiota, and PD.

Cyanidin-3-*O*-glucoside (Cy-3-G), a major water-soluble flavonoid anthocyanin, is mainly present in plant-based foods (including leafy vegetables, berries, red cabbages, and colored grains) and possess protective effects on many organs (Jia et al., 2020). It has a wide range of pharmaceutical benefits, such as antioxidant activities (Liu et al., 2018), gut microbiota modulation (Tian et al., 2019), neuroprotective effects (Bhuiyan et al., 2011), anticancer (Liang et al., 2019), and metabolic syndrome (Jiang et al., 2019). Furthermore, the previous researches set the foundation for the further study of Cy-3-G to PD. Therefore, this study takes the “imbalance” and “balance” of intestinal microecology as the starting point to investigate the “gut-to-brain” hypothesis using metabolomics combined with 16S rRNA gene sequencing methods. Finally, the metabolic biomarkers and gut microbiota biomarkers of PD were screened and identified, and the research system of “Cy-3-G-gut microbiota—PD” was constructed to explore the relationship between its treatment of PD and intestinal microecological “imbalance” and “balance”.

## Materials and Methods

### Chemicals and Reagents

Liquid chromatography-mass spectrometry (LC-MS) grade methanol, formic acid, and ammonium acetate were purchased from Thermo Fisher Scientific (Waltham, MA, United States). 1-methyl-4-phenyl-1,2,3,6-tetrahydropy ridine (M0896, MPTP) was obtained from Aladdin-e (Shanghai, China). Cy-3-G (HY-N0640) and dimethyl sulfoxide (DMSO, HY-Y0320) were purchased from MedchemExpress (Monmouth Junction, NJ, United States). Ultra-high purity water was prepared by the Millipore-Q water purification system (Millipore, Bedford, MA, United States). HiPure Stool DNA Kits were obtained from Guangzhou Magen Biotechnology Co., Ltd. (Magen, Guangzhou, China). Agarose and goldview were provided by Beijing Mengyimei Biotechnology Co. (Beijing, China). Anhydrous alcohol was supplied by Guangzhou Chemical Reagent Factory (Guangzhou, Beijing). MinElute PCR Purification Kit was bought from New England Biolabs, Inc. (Beverly, MA, United States). AMPure XP magnetic beads were purchased from Beckman Coulter (United States).

### Rats and Treatments

A total of 35 specific pathogen free (SPF) male C57BL/6J mice (7–8 weeks old, 20–25 g, animal license No. SCXK (Su) 2018-0008) were bought from GemPharmatech Co. During the whole experiment, the mice were housed in a temperature- and humidity-controlled holding facility (22 ± 2°C, 55% ± 5% humidity) with a 12-h light/dark cycle and free access to food and water. After 3 days of adaptation, 35 mice were randomly divided into three groups, namely, control, model, and Cy-3-G (10, 20, and 40 mg/kg), each with 7 mice. All procedures conformed to the principles of the Care and Use of Laboratory Animal and were approved by the Animal Ethics Committee of the Nanjing University of Chinese Medicine.

**FIGURE 1:**
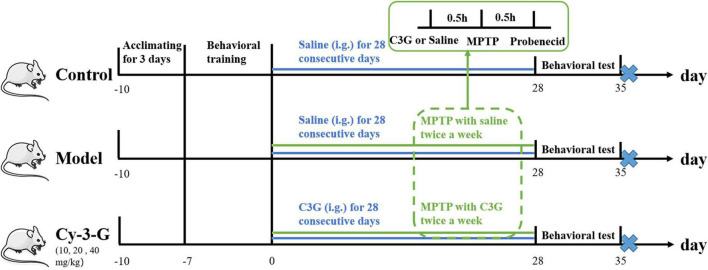
Schematic representation of the experiment.

The five groups of mice were pretrained for behavioral tests in the 1st week. In the next 4 weeks, the mice in the control and model groups were given an equal volume of normal saline, and the Cy-3-G group received 10, 20, and 40 mg/kg Cy-3-G. Then, the mice of the PD model group (model group and Cy-3-G group) were made by subcutaneously injecting MPTP with a dose of 20 mg/kg/d two times a week for 0.5 h after the intraperitoneal injection of Cy-3-G. The control group was given an equal volume of saline. Simultaneously, mice of the model and Cy-3-G groups received 250 mg/kg probenecid (intraperitoneal injection) at 0.5 h after the injection of MPTP. The control group received an equal volume of DMSO. All the mice were sacrificed on the 36th day after the behavioral test, and the experimental procedures are shown in Figure 1.

### Sample Collection and Preparation

After the mice were sacrificed, blood samples were collected and centrifuged at 4,500 rpm for 10 min at 4°C to obtain supernatants for metabolomic analysis. At 4°C, 100 μl of serum was added to 400 μl of 80% (v/v) methanol solution, vortexed, and centrifuged at 15,000 × *g* for 20 min. Some of the supernatants were diluted to a final concentration containing 53% methanol using LC-MS grade water. The samples were subsequently transferred to a fresh Eppendorf tube and then centrifuged at 15,000 × *g*, 4°C for 20 min for LC-MS/MS analysis.

At least 2 fecal pellets were received from each mouse on the 36th day after the behavioral test. They were placed in sterile EP tubes and stored at −80°C for 16S rRNA gene sequencing analysis.

### Effects of Cy-3-G on the Behavioral Test of MPTP-Intoxicated Mice

#### Rotarod Test

The rotarod test was used to evaluate the mouse motor coordination and balance based on the rotarod apparatus (Med Associates, United States). The method was performed in the same way as in the previous research (Zhu et al., 2021). The rotarod was linearly accelerating from 5 to 40 rpm in 5 min. Afterward, the mice were placed in the rotarod to conduct an experiment, and the mean latency time to fall of the rotarod was recorded.

#### Open-Field Test

The open-field test (OFT) was used to evaluate locomotor activity and anxiety behavior and was performed according to previous research (Zhang et al., 2017). The mice were placed directly in the box, which was divided into 24 grids of 8 cm × 8 cm. Afterward, the mice were removed from the box and placed in the center of the open field and were video recorded for 5 min (ambulatory movements and rearing) after being preadapted for 5 min. The box was cleaned using 10% alcohol between the tests. Furthermore, the research was scored on the distance traveled and the amount of rearing in 5 min.

#### Forced Swimming Test

To assess the limb coordination ability and motility of the mice, we utilized the forced swimming test (FST) (Sun et al., 2021). The mice were removed from mouse cages and placed in a glass tank (20 cm × 30 cm × 20 cm) with a water depth and temperature of 15 cm and 25°C, respectively. We were recording the immobility during the last 1 min. When the mice floating were motionless or making the necessary movements to keep their heads above the water, it was considered immobile. The FST was repeated 3 times, and the averages were calculated.

#### Pole Test

The pole test was performed to assess the motor coordination and bradykinesia ability and was conducted according to the previously described method (Luo et al., 2018). The apparatus consisted of a plastic rod (diameter: 0.5 cm, height: 50 cm) and a wooden ball on the pole. The mouse was placed on the top of the pole, and the time of turning its head downward and the total time from its placement to the bottom with its hind limbs contacting the ground were recorded. The pole test was repeated three times with an interval of 2 min, and the average times were analyzed.

#### Tail Suspension Test

The tail suspension test (TST) was used to assess depression-like behavior in MPTP-induced mice. Mice were suspended, acoustically and visually isolated, 50 cm above the floor. In this study, the mice did not touch the tail suspension instrument except for the tail, and the immobility time was recorded during a 5-min test.

### Immunohistochemistry

For immunohistochemistry, tissues (brains and intestines) were sectioned at 30 μm thickness and treated with methanolic H_2_O_2_ for 30 min. The sections were incubated with 0.5% Triton X-100 in phosphate-buffered saline (PBS) for 15 min and blocked with 4% normal serum in PBS for 15 min before incubating with the primary antibody. The sections were incubated overnight with TH (1:2,000, Servicebio, GB11181), ZO-1 (1:300, Servicebio, GB111981), occludin (1:500, Servicebio, GB111401), or claudin (1:300, Servicebio, GB11032). The sections were washed three times in PBS (pH 7.4, and incubated with secondary antibodies (1:200, Servicebio, GB23303) for 50 min at room temperature. After the slices were slightly spin-dried 3 times, 3,3′-diaminobenzidine tetrahydrochloride (DAB) condensed chromogen (Servicebio, G1211) was utilized to visualize. After immunostaining, sections were counterstained with hematoxylin (Servicebio, G1004). Sections were photographed using the Vectra Polaris Imaging System (Akoya).

### Metabolite Extraction and UHPLC-MS/MS Analysis

#### Instrument Condition

The serum was analyzed using a Vanquish UHPLC liquid chromatography system coupled with a Q Exactive*™* HF-X Hybrid Quadrupole-Orbitrap Mass Spectrometer (Thermo Fisher, Bremen, Germany) with a heated electrospray ionization source (ESI). A Hypesil Gold column (100 mm × 2.1 mm, 1.9 μm) and 17 min linear gradient at a flow rate of 0.2 ml/min were utilized. The mobile phase was comprised of A (methanol) and B (0.1% formic acid in water) for the positive mode and A (methanol) and B (5 mM ammonium acetate, pH 9.0) for the negative mode. The solvent gradient was set as follows: 2% A, 1.5 min; 2–100% A, 12.0 min; 100% A, 14.0 min; 100–2% A, 14.1 min; 2% A, 17 min. The mass spectrometer of Q Exactive*™* HF-X was used in the ESI positive/negative mode with a spray voltage of 3.2 kV, a capillary temperature of 320°C, a sheath gas flow rate of 40 arb, and an aux gas flow rate of 10 arb.

#### Data Processing and Metabolite Identification

Raw data files acquired through UHPLC-MS/MS were processed using Compound Discoverer 3.1 software (CD3.1, Thermo Fisher) for realizing peak alignment, peak picking, and quantitation for each metabolite. All normalized data were entered into R package models^1^ for multivariate statistical analysis, such as unsupervised dimensionality reduction method principal component analysis (PCA) and orthogonal projection to latent structures-discriminant analysis (OPLS-DA). A cross-validation and permutation test (200 permutations) were utilized to further validate the OPLS-DA model. For the cross-validation and permutation test, the data were partitioned into seven subsets, where each of the subsets was then utilized as a validation set. Among them, the correlation coefficient (R^2^) explains the total variation in the data matrix, which was shown in the model. Predictive ability (Q^2^) values were usually recognized as the strongest statistical parameter for model reliability research in metabolomics. Variable importance in projection (VIP) score of the OPLS model was performed to arrange the significant metabolites that were screened between two groups, with a threshold of 1. Furthermore, multivariate analysis methods based on the *T*-test were utilized for screening differential metabolites. Taken together, variables with a *p*-value of *T*-test < 0.05 and VIP ≥ 1 were considered potential biomarkers associated with PD or Cy-3-G. Volcano plot analysis combined with fold changes in abundance was utilized to illustrate the regulation of differential metabolites. Pathway enrichment of potential biomarkers was executed using MetaboAnalyst 5.0^2^, which was based on the Kyoto Encyclopedia of Genes and Genomes (KEGG) database. The pathway was perceived as markedly enriched, with a threshold of 0.5.

#### 16S rRNA Gene Sequencing Analysis

According to the standardized protocol, HiPure Soil DNA Kits were utilized for extracting microbial genomic DNA. The V3 + V4 target region of the 16S rRNA gene was amplified using PCR with the universal primers (341F: 5′-CCTACGGGNGGCW GCAG-3′ and 806R: 5′-GGACTACHVGGG TATCTAAT-3′). According to the standard protocols, the samples were pooled in equimolar and paired-end sequenced (PE250) on an Illumina Novaseq 6000 platform. The raw reads were stored in the NCBI Sequence Read Archive (SRA) database. Furthermore, the following steps were utilized to make the quality control of reads and the effect of merging. First, raw reads were filtered using FASTP (Chen et al., 2018) (version 0.18.0) according to the following rules: reads that included more than 10% of unknown nucleotides (N) or less than 50% of bases with a quality (Q-value) > 20 were removed. Second, FLSAH (Magoč and Salzberg, 2011) (version 1.2.11) software coupled with a minimum overlap of 10 bp and mismatch error rates of 2% was utilized to merge the paired-end clean reads into raw tags. Ultimately, raw tags whose base number in the continuous low quality value (the default quality threshold is ≤ 3) reached 3 bp default length or less than 75% of the tag length were broken. The clean tags were clustered at a 97% sequence similarity level using the UPARSE (Edgar, 2013) (version 9.2.64) pipeline to obtain operational taxonomic units (OTUs). Effective tags were obtained by removing all chimeric tags using the UCHIME algorithm (Edgar et al., 2011). It was selected as a representative sequence among the clusters in which the tag sequence owns the highest abundance. The tag sequence with the highest abundance was selected as a representative sequence within each cluster.

The characteristic OTU sequences were classified using a naive Bayesian model with an RDP classifier based on the SILVA database, with confidence threshold values ranging from 0.8 to 1. The LEfSe software in the R project was utilized to screen the biomarkers in each group. The Welch’s *t*-test and Wilcoxon signed-rank test were utilized to calculate the alpha index between groups. Beta diversity analysis, principal coordinate analysis (PCoA), for example, was performed using the R project Vegan package and plotted in the R project ggplot2 package to compare the similarity of species diversity.

### Integrated Microbiome and Metabolomic Analysis

To evaluate the possible relationships between gut microbiota and metabolites, the correlation analysis of these phylum/genera and differential metabolites were conducted. The phylum/genus-metabolite networks were built based on the Gephi software (version 0.9.2) for visualization (Zuo et al., 2021), with | r| > 0.8 and *p* < 0.05 as the criterion. The variables in the established networks were analyzed for metabolite set enrichment analysis (MSEA) based on the MetaboAnalyst 5.0 database. Among them, metabolite was considered significant when *p* < 0.05 was used as the standard.

## Results

### Cy-3-G Treatment Attenuates MPTP-Induced Dopaminergic Neurodegeneration and Intestinal Barrier Destruction

**FIGURE 2:**
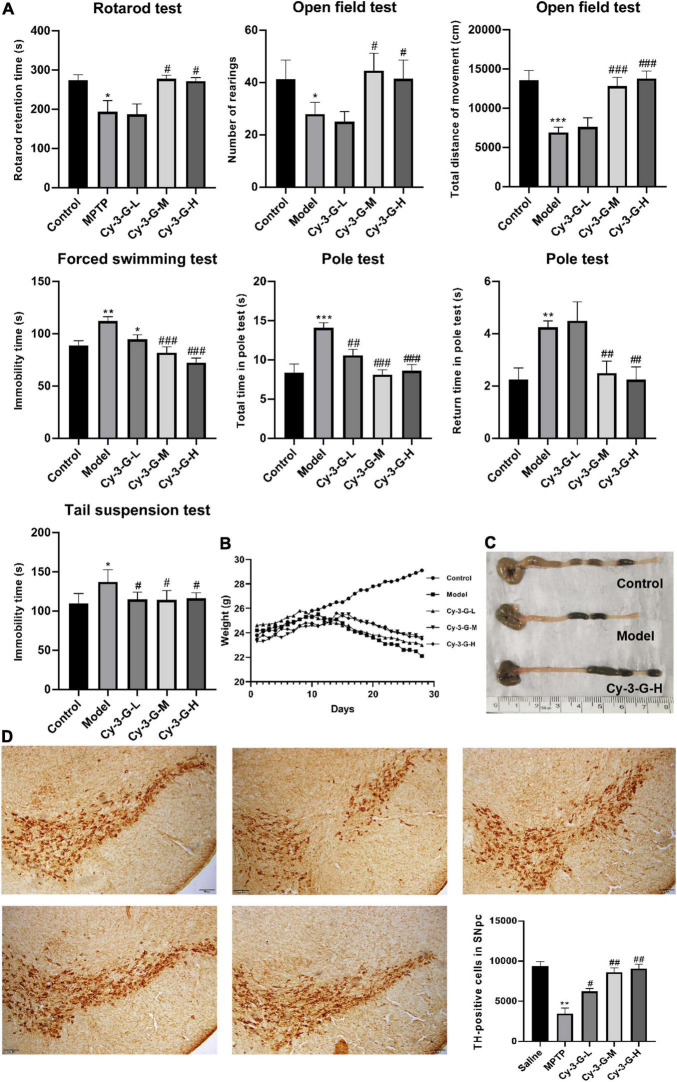
Effects of Cy-3-G on the behavioral test **(A)**, body weight **(B)**, and colon length **(C)** of PD model mice; Representative microphotographs of dopaminergic neurons stained for TH and the quantification of TH positive cells in each group **(D)**. The pictures were taken at an original magnification of 10×. Control: blank group; Model: MPTP -intoxicated group; Cy-3-G-H: high dosage group; Cy-3-G-M: medium dosage group; Cy-3-G-L: low dosage group; *p < 0.05, **p < 0.01, ***p < 0.001 vs. Control; ^#^*p* < 0.05, ^##^*p* < 0.01, ^###^*p* < 0.001 vs. Model.

There was a significant variation in the mice’s weight and the appearance of colonic tissue between the groups during the study, showing a difference in the toxicity of MPTP in different groups (Figures 2B,C). Simultaneously, to evaluate the pharmacological action of Cy-3-G on MPTP-induced behavioral deficits, a series of behavioral tests, including the rotarod test, OFT, FST, pole test, and TST, were established. As shown in Figure 2A, MPTP-induced mice have a significantly longer rod-standing time compared with control group mice, whereas Cy-3-G treatment significantly improves MPTP-intoxicated mice to stand for a shorter time. Moreover, the OFT results showed that MPTP-intoxicated mice significantly decreased the distance traveled compared with the control group, while Cy-3-G treatment group mice showed significantly decreased these phenomena. Among the MPTP treatment group mice, the time of turning their heads downward and the total time from their placement to the bottom significantly increased compared with the control group. Cy-3-G could improve the parameter at high and middle doses. According to the TST results, the immobility time in model mice was prolonged significantly compared with the control group, and Cy-3-G could improve obviously. Furthermore, we found that there were no differences in FST between the MPTP-treated group and control group. The results showed that treatment with Cy-3-G could significantly improve the MPTP-induced behavioral deficits in mice.

**FIGURE 3:**
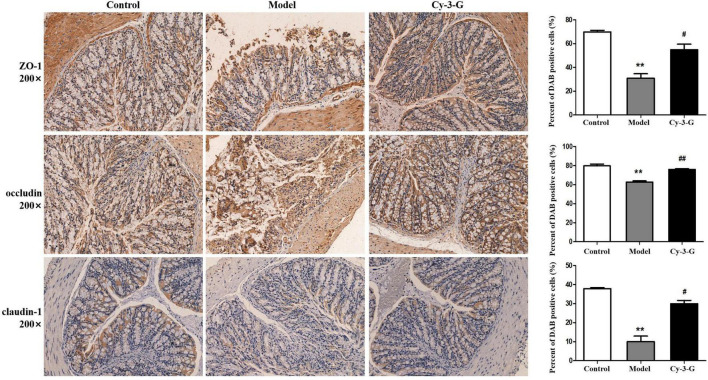
Location and expression of tight junction protein ZO-1, occludin, and claudin-1 in colonic epithelium from different groups, respectively. **p < 0.01 vs. control; ^#^p < 0.05, ^##^*p* < 0.01 vs. model.

To define the neuroprotective effect of Cy-3-G against MPTP-induced neurotoxicity, TH-positive cells in the substantia nigra pars compacta (SNpc) were quantified. Compared with the control group, the number of TH-positive cells was notably changing, while the change trends of the Cy-3-G-treated group were contrary to that of the model group (Figure 2D). Control, model, and Cy-3-G-treated group mice were subjected to immunohistochemical experiments. As a result, the expressions of the tight-junction proteins (ZO-1, occluding, and claudin) were significantly lower in the MPTP model group than in the control group, while such effects were abolished by treatment with Cy-3-G (Figure 3). The results illustrated that treatment with Cy-3-G effectively increased the expression of intestinal tight-junction proteins and improved the disorder of gut microbiota.

### Serum Metabolic Profiling of Cy-3-G in Treating Parkinson’s Disease Mice

**FIGURE 4:**
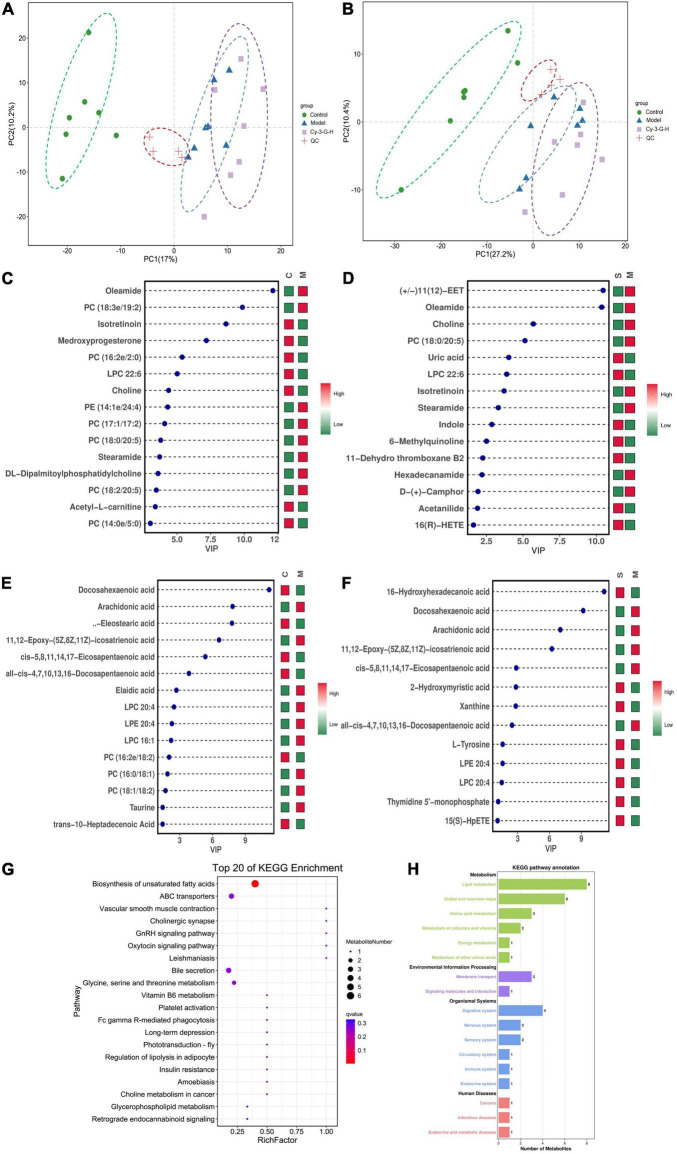
PCA score plot of all analyzed samples in positive-ion **(A)** mode and negative-ion **(B)** mode with the statistical parameters; VIP of OPLS-DA between the control and model groups in positive-ion **(C)** mode and negative-ion **(D)** mode; VIP of OPLS-DA between the model and Cy-3-G-H groups in positive-ion **(E)** mode and negative-ion **(F)** mode; Bubble chart of GO function analysis **(G)** and the metabolites statistics of KEGG **(H)**.

**FIGURE 5:**
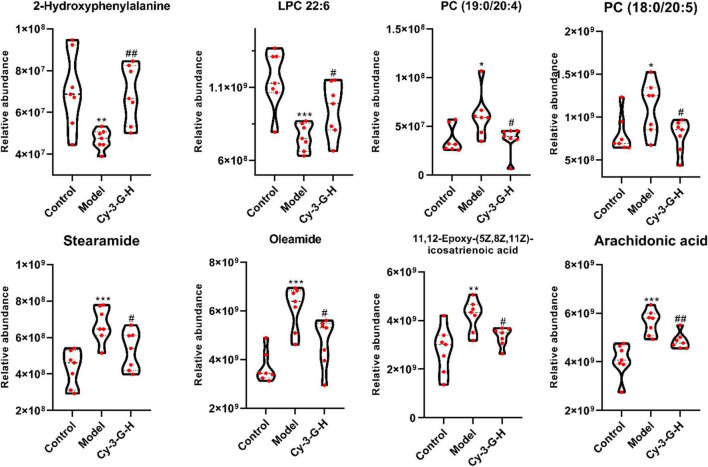
Variations in the trends of the metabolites that are biomarkers of both PD and Cy-3-G treatment. *p < 0.05, **p < 0.01, ***p < 0.001 vs. Control; ^#^*p* < 0.05, ^##^*p* < 0.01 vs. Model.

Serum metabolic profiling of control, model, and Cy-3-G-treated mice was obtained using LC-MS/MS. Based on the serum metabolic profiling, the PCA model results showed that the control group, MPTP-intoxicated group, and Cy-3-G-treated group could be obviously separated (Figure 4). In the OPLS-DA model, a significant difference between control and model mice was found, indicating that Cy-3-G results in remarkable disease changes. Variables with *p* a value of *T*-test <0.05 and VIP ≥ 1 were considered potential biomarkers associated with PD or Cy-3-G (Supplementary Figure 1 and Figures 4C–F), which were screened for further structure identification. Furthermore, a permutation test (200 permutations, Supplementary Figure 1) was utilized to check for overfitting of the OPLS-DA. We identified the screened potential biomarkers using the retention time, accurate masses, and fragment ions in MS spectra acquired through LC-Q Exactive Orbitrap MS. Simultaneously, the information was further verified using online databases such as HMDB^3^, METLINE^4^, and MassBank^5^. Interestingly, forty-one metabolites in the positive model and twenty-six metabolites in the negative model (Supplementary Table 1) were identified as potential biomarkers of MPTP-induced PD. Concurrently, twenty-three metabolites in the positive model and thirteen metabolites in the negative model (Supplementary Table 2) were identified as potential biomarkers of Cy-3-G treatment in PD. These metabolic biomarkers were mainly involved in lipid metabolism, amino acid metabolism, cofactor metabolism, and vitamin and energy metabolism (Figures 4G,H). Through comparison, a total of 8 biomarkers (2-hydroxyphenylalanine, LPC 22:6, PC (19:0/20:4), PC (18:0/20:5), stearamide, oleamide, 11,12-Epoxy-(5Z,8Z,11Z)-icosatr-enoic acid, arachidonic acid) were simultaneously altered with opposing trends in variation after MPTP and Cy-3-G treatment (Figure 5).

### Identification and Functional Annotation of Differential Gut Microbiota

**FIGURE 6:**
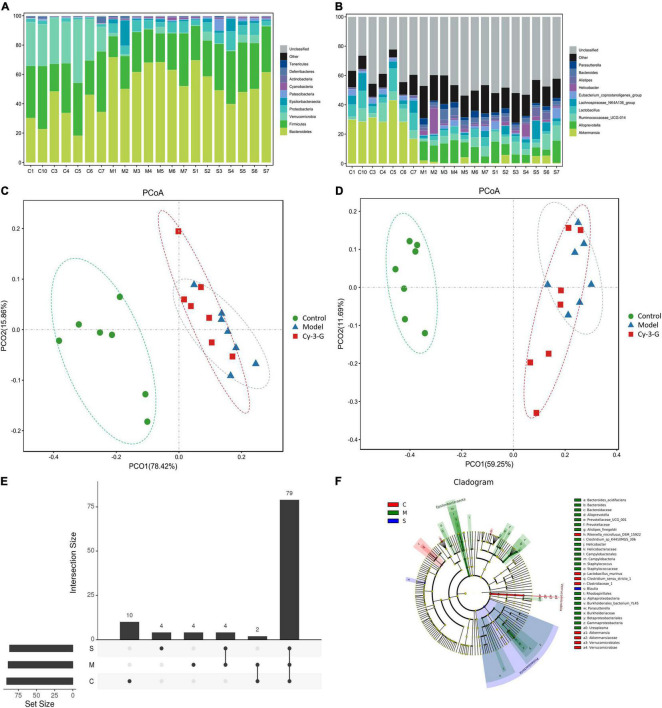
The histogram of species distribution at the phylum **(A)** and genus **(B)** levels in three groups revealed by 16S rRNA sequencing (different colors represent different bacteria at phylum or genus levels). PCoA analysis among control, model, and Cy-3-G groups at the phylum **(C)** and genus **(D)** levels. Upset plot based on the gut microbiota among control, model, and Cy-3-G groups **(E)**; Cladogram of microbial taxa differentially represented among control, model, and Cy-3-G group **(F)**.

In 21 samples, 3,043 OTUs were obtained. The species annotation analysis results at the phylum and genus levels are shown in Figures 6A,B. The figure demonstrated ten abundances of bacteria at phylum and genus level that ranked in the top ten, and the other bacteria were grouped together as “Other,” while unclassified exemplified bacteria were those that were not taxonomically annotated. As shown in Figures 6A,B, Bacteroidetes, Firmicutes, and Verrucomicrobia were the most dominant phyla in the gut microbiota of all fecal samples at the phylum level obtained in the result. During modeling, the abundances of Bacteroidetes were significantly increased in the MPTP model group compared with the control group, while the abundances of Firmicutes and Verrucomicrobia were significantly decreased. By contrast, the change trends of the Cy-3-G-treated group were contrary to that of the model group. These results were consistent with the previous reports that Firmicutes and Bacteroidetes imbalances were related to the changes in age (Mariat et al., 2009), including PD. In previous studies, the Verrucomicrobia phylum (Akkermansia genus or species, for example) was significantly changed in patients with PD (Hill-Burns et al., 2017).

**FIGURE 7:**
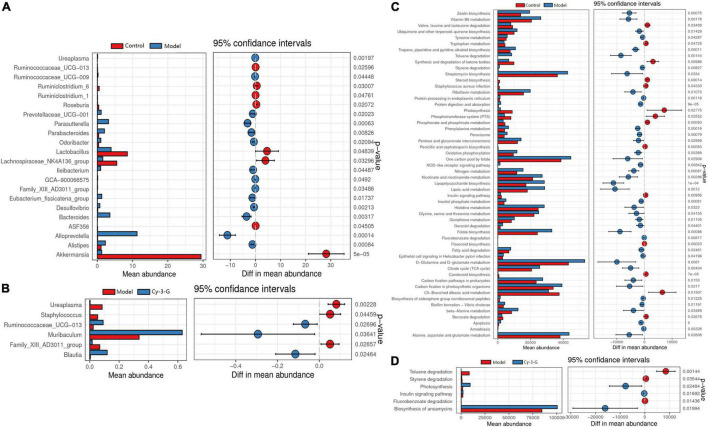
The differential abundance taxa in phyla **(A)** and genera **(C)** based on the Welch’s *t*-test analysis between control vs. model **(A)** and model vs. MPTP-induced **(C)** groups; The function of the gut microbiota of mice in the control and MPTP-induced groups based on Tax4Fun between control vs. model **(B)** and model vs. MPTP—induced **(D)** groups.

**FIGURE 8:**
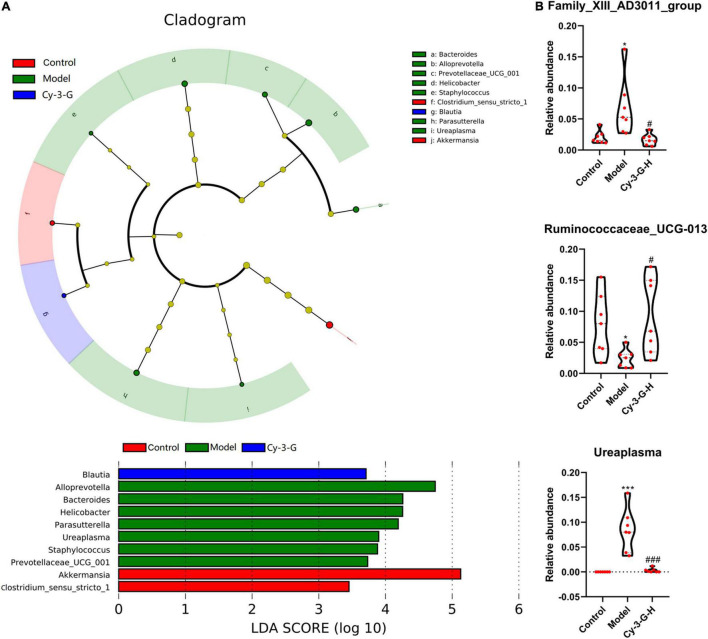
Variations in the trends of the taxa in genera that are biomarkers of both PD and Cy-3-G treatment based on cladogram and LDA **(A)** and *t*-test analysis **(B)**. *p < 0.05, ***p < 0.001 vs. control; ^#^*p* < 0.05, ^###^*p* < 0.001 vs. model.

The principal coordinate analysis (PCoA) results showed that gut microbiota composition profiles of the control, MPTP-induced, and Cy-3-G-treated groups were separated evidently (Figures 6C,D). An Upset plot was performed using the R project UpSetR package to identify unique and common species (Figure 6E) in order to illustrate the characteristics of species composition among groups. In the control, MPTP-induced, and Cy-3-G-treated groups, 10, 4, 4 unique species were screened, respectively. Simultaneously, 4 and 2 common species were found in two groups, namely, control vs. MPTP-induced groups and MPTP-induced vs. Cy-3-G-treated groups. Afterward, 79 common species among the three groups were identified. It was deemed that there are no such species in the group when the average tag number of a species in the group is less than 1. LDA–LEfSe analysis [linear discriminant analysis (LDA) integrated with effect size] was utilized to generate a cladogram in order to identify the distinguishing taxa within the groups (Figure 6F). Welch’s *t*-test analysis was utilized to identify taxa (phyla and genera) based on the differential abundances within the groups. Five differential phyla were recognized with a P-value of < 0.05, including Bacteroidetes, Proteobacteria, Verrucomicrobia, Tenericutes, and Cyanobacteria (Figures 7A,C). Compared with the control group, the abundance of Proteobacteria and Bacteroidetes was significantly increased, whereas the abundance of Verrucomicrobia, Tenericutes, and Cyanobacteria decreased in the MPTP-induced group. At the generic levels, twenty-two differential genera were recognized with a P-value of < 0.05 (Figure 7A) in the MPTP-induced model group compared with the control group. Simultaneously, the significantly changed microbes in abundance, such as Muribaculum, Ruminococcaceae_UCG-013, Blautia, Family_XIII_AD3011_group, Ureaplasma, and Staphylococcus, were shown in the Cy-3-G group compared with the MPTP-induced group (Figure 7C). Through comparison, a total of 12 biomarkers (Family_XIII_AD3011_group, Ruminococcaceae_UCG-013, and Ureaplasma based on the Welch’s *t*-test analysis in Figure 8A; Akkermansia, Alloprevotella, Helicobacter, Bacteroides, Parasutterella, Prevotellaceae_UCG_001, Clostridium_sensu_stricto_1, Blautia, Ureaplasma, and Staphylococcus based on the LDA-LEfSe analysis in Figure 8B) were simultaneously altered with opposing trends in variation after MPTP and Cy-3-G treatment.

Tax4Fun was utilized to predict the function of the gut microbiota of mice in control and MPTP-induced groups. The results showed that the differential microbes were closely related to amino acid metabolism, NOD-like receptor signaling pathway, carbohydrate metabolism, cofactor metabolism, and vitamin and energy metabolism (Figures 7B,D).

### Correlation Analysis for Differential Metabolites and Microbes

**FIGURE 9:**
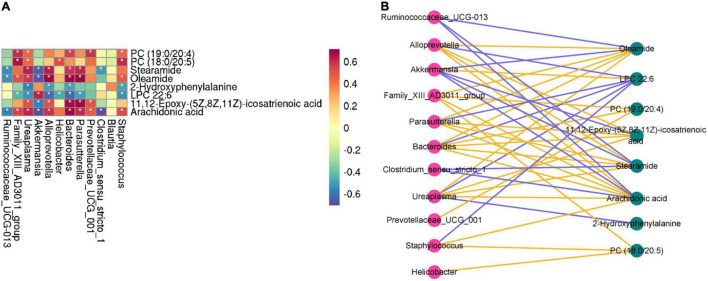
Correlation heatmap is applied to represent the correlation values between perturbed gut microbe genus and altered fecal metabolites **(A)**. “*” (white) refers to the significance level of the corresponding correlation coefficient in each group. *P < 0.05. Interaction network diagram of the metabolites–microbes pathways based on correlation analysis **(B)**. The yellow (blue) line represents a positive correlation (negative correlation) between the metabolite and microbes.

A correlation heatmap and network diagram were utilized to delegate the covariation between perturbed gut microbe genus and altered metabolites (Figure 9A). Moreover, the gut microbiota (Family_XIII_AD3011_group, Ruminococcaceae_UCG-013, Ureaplasma, Akkermansia, Alloprevotella, Helicobacter, Bacteroides, Parasutterella, Prevotellaceae_UCG_001, Clostridium_ sensu_stricto_1 and Staphylococcus) and differential metabolites (2-hydroxyphenylalanine, LPC 22:6, PC (19:0/20:4), PC (18:0/20:5), stearamide, oleamide, 11,12-epoxy-(5Z,8Z,11Z)-icosatrienoic acid, and arachidonic acid) were significantly correlated, demonstrating that the changes in serum metabolites may be associated with the gut microbiota disruptions. Simultaneously, we found that the metabolites of LPC 22:6 and gut microbiota (except Akkermansia, Clostridium_sensu_stricto_1, and Blautia) showed negative correlations. The metabolite of 2-hydroxyphenylalanine has a negative correlation with the gut microbiota (Alloprevotella, Bacteroides, Ureaplasma, and Staphylococcus). Akkermansia and Clostridium_sensu_stricto_1 have a negative correlation with gut microbiota. As for other aspects, there is a positive correlation between metabolites and gut microbiota. Concurrently, the interaction network diagram of the metabolites–microbes was constructed to further study the intrinsic relationship between these metabolites and gut microbiota (the solid-line curve represents positive correlation and the dotted line represents negative correlation in Figure 9B).

## Discussion

### The Research of Parkinson’s Disease From the Perspective of Metabolic Pathways

Studies have revealed that both dysbiosis and alteration of gut microbiota could affect the CNS, thereby causing CNS diseases (PD included) (Sun and Shen, 2018). However, the correlation between gut microbiota and metabolomics remains largely unidentified. The findings of this study suggest that MPTP-induced PD is related to lipid metabolism, amino acid metabolism, cofactor metabolism, and vitamin and energy metabolism. The 16S rRNA sequencing results showed that the differential gut microbiota were interrelated with amino acid metabolism, NOD-like receptor signaling pathway, carbohydrate metabolism, cofactor metabolism, and vitamin and energy metabolism. The correlation between differential gut microbiota and potential biomarkers was analyzed.

Choline is necessary for the synthesis of the neurotransmitter acetylcholine and phospholipid (an integral component of cell membranes) (Amo et al., 2019). Some studies have revealed mitochondrial respiratory chain dysfunction and aberrant choline metabolism using SH-SY5Y cells (Baykal et al., 2008). Betaine is a natural product that is widely distributed in plants, animals, microorganisms, and foods such as *Lycium chinense* (Shin et al., 1999; Craig, 2004). Previous studies have revealed that betaine could maintain the appropriate balance between phosphatidyl ethanolamine and phosphatidyl choline to preserve the proper membrane flexibility and prevent lipid peroxidation through its existence among the methylation within the cellular membranes (Alirezaei et al., 2015). It is shown that betaine has a protective effect against stress-induced oxidative damage (Ganesan and Anandan, 2009). However, the increased reactive oxygen (ROS) production and oxidative damage to biological molecules are related to various pathological mechanisms, such as neurodegenerative disorders (Batandier et al., 2002). A variety of studies have shown that the mechanism of action of rotenone (a model drug of PD), the inhibition of complex I, leads to the growth of mitochondrial oxidative stress. Several *in vitro* studies have demonstrated that betaine has protective effects on rotenone-induced PC12 cells. Phenylalanine, an essential amino acid, is derived from animal protein and vegetables and is acquired exclusively through the diet. At the same time, phenylalanine is easily absorbed by the brain and then processed into neurotransmitters. In the long run, normal brain functioning could be interfered with by phenylalanine. The biosynthesis of L-3,4-dihydroxyphenylalanine (L-DOPA) relies on the precursor molecule phenylalanine.

According to the result, five metabolites (docosapentaenoic acid, eicosapentaenoic acid, docosahexaenoic acid, *cis-*5,8,11,14,17-eicosapentaenoic acid, and adrenic acid) were decreased in the MPTP-induced group, leading to the metabolic disorder of lipid metabolism. Docosapentaenoic acid, eicosapentaenoic acid, and docosahexaenoic acid are omega-3 polyunsaturated fatty acids that are beneficial to treat neurodegenerative diseases such as Parkinson’s disease, Alzheimer’s disease, and Huntington’s disease (Shirooie et al., 2018). Simultaneously, polyunsaturated fatty acids also have reliable neuroprotective effects using the PD animal models and then in clinical trials, mainly through reducing apoptotic cell death and activating the glutathione-dependent antioxidant systems, which are conducive to a decrease in motor dysfunction.

### The Research of Parkinson’s Disease Based on the Correlation Analysis for Differential Metabolites and Microbes

In the Verrucomicrobia phylum, *Akkermansia muciniphila* possesses a beneficial influence on the mucous membrane and improves the barrier function of the gut epithelium (Wang et al., 2021). Thus, the maintenance of a health gut barrier and other pathogenic factors of many bacteria, including lipopolysaccharide and bacterial endotoxin, could therefore injure the host. In contrast, *Akkermansia* could degrade the mucus layer by using mucus as an energy source (Kumari et al., 2013). *Akkermansia* was also associated with a high prediction score for PD (Wang et al., 2021). The phenomenon of Firmicutes/Bacteroides ratio is found to vary with age (Alirezaei et al., 2015), and relevance with neurodegeneration (PD, for example) was considered. Several members of the Lachnospiraceae family, such as Blautia, are gradually attracting attention as a result of their ability to produce short-chain fatty acids (SCFAs). These metabolites (such as acetate, propionate, and butyrate) appear to play an important role in coordinating the function of the enteric nervous system and promoting gastrointestinal integrity and motility (Zhang et al., 2016). Simultaneously, the potential role of SCFA-producing bacteria has been highlighted in the pathogenesis of PD with respect to their contribution to the reduction of SCFAs to the development of gastrointestinal motility dysfunctions (Vascellari et al., 2020). At the genus level, helicobacter was found to be significantly increased. It has been reported that the intestinal bacteria of helicobacter could cause the misfolding of a-syn and aggravate the inflammation effect of a-syn by activating innate immunity (Stolzenberg et al., 2017). From these results, we suggest that the misfolded a-syn could activate the proinflammatory factors secrete and microglia-mediated natural immune response, and enhance the differentiation and multiplication of T cells into effector T cells. Neurotoxicity (apoptosis and death of dopamine neurons) could lead to the occurrence of PD and may occur as a result of the effector T cells crossing the blood–brain barrier and migrating to brain lesions (Fang, 2016).

In conclusion, this study illustrated that PD is associated with gut microbiota disorders and that Cy-3-G could play a role in the treatment of PD by modulating the structure and metabolism of gut microbiota. Nevertheless, the following works need to be done if we want to get more comprehensive and reliable outcomes. First, the metagenomic experiment should be established to analyze the function of these differential gut microbiotas. Second, the sample size should be expanded and clinical data should be collected for more in-depth study in order to better guide the clinical work. Finally, the optimal dosage of Cy-3-G in the treatment of PD’s gut microbiota dysbiosis-integrated pharmacology should be illustrated.

## Conclusion

Although a range of biomarkers derived from clinical, neuroimaging, and genetic studies have been proposed (Liepelt et al., 2009; Heywood et al., 2015; Majbour et al., 2016), an analysis must necessarily be viewed as more qualitative than quantitative. Therefore, sensitive, specific, and reliable biomarkers for PD remain elusive. However, since PD is a multifactorial disease, multiple mechanisms may likely contribute to its pathogenesis. Despite decades of research, the underlying etiopathogenesis of PD is still not fully elucidated. Given the lack of knowledge regarding the mechanisms that regulate the onset and progression of the disease pathology, new approaches dedicated to the discovery of specific biomarkers that offer more accurate diagnosis and better monitoring of PD progression and prognosis are in urgent need. This study targets the intestinal microbiota for the prevention of brain-related degenerative diseases. This study might be a supplement for the deficiency of the screening of diagnostic biomarkers and the basic research of Cy-3-G.

In the above work, serum metabonomics combined with 16S rRNA gene sequencing analysis were utilized to study the specific interaction between PD and gut microbiota and the inherent regulative mechanism of Cy-3-G on PD’s gut microbiota. Metabonomics was applied to screen the differential metabolites and metabolic pathways. The study found that forty-one metabolites in the positive model and twenty-six metabolites in the negative model were potential biomarkers of MPTP-induced PD. Concurrently, twenty-three metabolites in the positive model and thirteen metabolites in the negative model were identified as potential biomarkers of Cy-3-G-treated PD. These metabolites and metabolic pathways were mainly related to lipid metabolism, amino acid metabolism, cofactor metabolism, and vitamin and energy metabolism. In the 16S rRNA gene sequencing analysis results, PD was found to be related to twenty-two different gut microbiotas at the genus level. The 16S rRNA sequencing result shows that the differential gut microbiotas were interrelated with amino acid metabolism, NOD-like receptor signaling pathway, carbohydrate metabolism, cofactor metabolism, and vitamin and energy metabolism. Our findings provide a theoretical basis for further understanding the mechanism underlying the role of gut microbiota in regulating PD.

## Data Availability Statement

The data has been successfully deposited: (1) For 16S rRNA analysis, the datasets generated can be found in the NCBI Trace Archive NCBI Sequence Read Archive, SRA accession: PRJNA821099. (2) For metabolomics analysis, the datasets generated can be found in the metabolights repository, and the accession number is MTBLS4509.

## Ethics Statement

The animal study was reviewed and approved by Animal Ethics Committee of Nanjing University of Chinese Medicine.

## Author Contributions

RN-C, LF, and JJ-T designed the study. WW, WL, and SL-Y collected the samples and performed statistical analyses. GX-Z, YW-W, and WW supervised metabolomics analyses. GX-Z, KW, and WW drafted the manuscript. All authors read and approved the final manuscript.

## Conflict of Interest

The authors declare that the research was conducted in the absence of any commercial or financial relationships that could be construed as a potential conflict of interest.

## Publisher’s Note

All claims expressed in this article are solely those of the authors and do not necessarily represent those of their affiliated organizations, or those of the publisher, the editors and the reviewers. Any product that may be evaluated in this article, or claim that may be made by its manufacturer, is not guaranteed or endorsed by the publisher.

## Footnotes

1
http://www.r-project.org/

2
https://www.metaboanalyst.ca/

3
http://www.hmdb.ca/

4
http://metlin.scripps.edu

5
http://www.massbank.jp/
