# Supplementary material for: Multi-Omics Integration in Mice With Parkinson’s Disease and the Intervention Effect of Cyanidin-3-O-Glucoside
Source: Front Aging Neurosci. 2022 Apr 29;14:877078. doi: 10.3389/fnagi.2022.877078 (PMC9099026; doi:10.3389/fnagi.2022.877078)
Supplement: Supplementary file 1 [file Table_1.DOCX]

Supplementary Material

Table S1: The identification of the potential biomarkers of MPTP-induced PD

| No. | m/z | Rt min | Formula | P value | FDR | VIP | Name |
| --- | --- | --- | --- | --- | --- | --- | --- |
| Positive | | | | | | | |
| 1 | 282.27914 | 15.23 | C18H35NO | 8.42E-05 | 0.003227824 | 12.12410717 | Oleamide |
| 2 | 780.5921 | 15.587 | C45H82NO7P | 0.014482583 | 0.085669727 | 9.860982263 | PC (18:3e/19:2) |
| 3 | 301.21582 | 13.954 | C20H28O2 | 0.000275212 | 0.007483991 | 8.641387565 | Isotretinoin |
| 4 | 345.24207 | 14.186 | C22H32O3 | 0.010126711 | 0.071737959 | 7.177760342 | Medroxyprogesterone |
| 5 | 520.33984 | 14.637 | C26H50NO7P | 0.037546107 | 0.1507277 | 5.380264924 | PC (16:2e/2:0) |
| 6 | 568.33978 | 14.566 | C30H50NO7P | 0.000481325 | 0.00944203 | 5.014336335 | LPC 22:6 |
| 7 | 104.10707 | 1.349 | C5H13NO | 0.000445755 | 0.009394296 | 4.377662927 | Choline |
| 8 | 752.55914 | 16.352 | C43H78NO7P | 0.001826668 | 0.026377363 | 4.306963291 | PE (14:1e/24:4) |
| 9 | 756.55505 | 16.352 | C42H78NO8P | 0.007782781 | 0.059644402 | 4.081791652 | PC (17:1/17:2) |
| 10 | 808.58618 | 15.196 | C46H82NO8P | 0.045237238 | 0.166735448 | 3.78647175 | PC (18:0/20:5) |
| 11 | 284.29495 | 15.622 | C18H37NO | 0.000582689 | 0.010915717 | 3.709965581 | Stearamide |
| 12 | 734.57129 | 16.293 | C40H80NO8P | 0.036776216 | 0.1507277 | 3.592784846 | DL-Dipalmitoylphosphatidylcholine |
| 13 | 804.55481 | 15.973 | C46H78NO8P | 0.003219029 | 0.036670827 | 3.459282251 | PC (18:2/20:5) |
| 14 | 204.12303 | 1.456 | C9H17NO4 | 0.003153528 | 0.036666001 | 3.385839018 | Acetyl-L-carnitine |
| 15 | 538.38733 | 15.767 | C27H56NO7P | 0.009413233 | 0.068408233 | 3.020518446 | PC (14:0e/5:0) |
| 16 | 812.617 | 15.363 | C46H86NO8P | 0.042813093 | 0.161271606 | 2.972477609 | PC (19:1/19:2) |
| 17 | 162.11263 | 1.368 | C7H15NO3 | 0.000644554 | 0.011560835 | 2.954665299 | DL-Carnitine |
| 18 | 331.26312 | 15.392 | C22H34O2 | 4.44E-07 | 9.35E-05 | 2.713905996 | Docosapentaenoic acid |
| 19 | 494.32486 | 14.467 | C24H48NO7P | 0.018728411 | 0.098675318 | 2.564764784 | PC (14:1e/2:0) |
| 20 | 538.35077 | 13.692 | C26H52NO8P | 0.000481622 | 0.00944203 | 2.433107073 | PC (9:0/9:0) |
| 21 | 544.34045 | 14.603 | C28H50NO7P | 0.011380202 | 0.074282751 | 2.374802044 | PC (18:4e/2:0) |
| 22 | 854.57092 | 15.982 | C50H80NO8P | 0.011877372 | 0.075282894 | 2.252535437 | PC (20:4/22:6) |
| 23 | 424.34241 | 13.361 | C25H46NO4 | 0.002864327 | 0.034008834 | 2.22653086 | ACar 18:2 |
| 24 | 548.37177 | 15.26 | C28H54NO7P | 0.032906798 | 0.142258617 | 1.69317032 | PC (16:2e/4:0) |
| 25 | 813.68378 | 15.365 | C47H93N2O6P | 0.011842289 | 0.075282894 | 1.476706825 | SM (d15:1/27:1) |
| 26 | 794.60754 | 16.411 | C46H84NO7P | 0.010103633 | 0.071737959 | 1.346721797 | PC (16:1e/22:4) |
| 27 | 492.30899 | 14.403 | C24H46NO7P | 0.000175251 | 0.006155707 | 1.343718421 | LPC 16:2 |
| 28 | 303.23154 | 14.99 | C20H30O2 | 6.77E-05 | 0.00294145 | 1.30958841 | Eicosapentaenoic acid |
| 29 | 585.27112 | 16.068 | C33H36N4O6 | 0.007993434 | 0.060164867 | 1.261508493 | Bilirubin |
| 30 | 801.68463 | 14.354 | C46H93N2O6P | 0.002839953 | 0.034008834 | 1.246214274 | SM (d25:0/16:1) |
| 31 | 169.09485 | 1.273 | C8H12N2O2 | 0.01977251 | 0.103186459 | 1.21784404 | Pyridoxamine |
| 32 | 446.32666 | 13.136 | C27H44NO4 | 2.77E-07 | 7.78E-05 | 1.173575463 | ACar 20:5 |
| 33 | 160.13353 | 1.454 | C8H17NO2 | 0.00612485 | 0.05192846 | 1.162120913 | Acetyl-methylcholine |
| 34 | 815.69202 | 15.703 | C47H95N2O6P | 0.004784454 | 0.046359705 | 1.12600329 | SM (d26:1/16:0) |
| 35 | 824.61731 | 15.725 | C47H86NO8 P | 0.027968186 | 0.126759036 | 1.114504811 | PC (19:0/20:4) |
| 36 | 165.05496 | 1.569 | C9H11NO3 | 0.006366913 | 0.052620667 | 1.105037745 | 2-Hydroxyphenylalanine |
| 37 | 296.06595 | 1.349 | C12H13ClF3NO2 | 0.002179742 | 0.028711293 | 1.103439694 | N-[2-chloro-6-(trifluoromethoxy)phenyl]-2,2-dimethylpropanamide |
| 38 | 482.32465 | 14.579 | C23H48NO7P | 0.029917684 | 0.132983487 | 1.09092043 | LPC 15:0 |
| 39 | 118.08643 | 1.513 | C5H11NO2 | 0.049459807 | 0.176585764 | 1.054128841 | Betaine |
| 40 | 550.3877 | 15.444 | C28H56NO7 P | 0.004559775 | 0.045222243 | 1.035309229 | PC (18:1e/2:0) |
| 41 | 170.09651 | 7.033 | C12H11N | 1.49E-09 | 6.29E-07 | 1.004657042 | Diphenylamine |
| Negative | | | | | | | |
| 42 | 327.23291 | 14.635 | C22H32O2 | 1.77698E-06 | 9.75118E-05 | 11.17401481 | Docosahexaenoic acid |
| 43 | 303.23273 | 14.694 | C20H32O2 | 0.000414957 | 0.004411418 | 7.833823829 | Arachidonic acid |
| 44 | 277.21719 | 14.46 | C18H30O2 | 1.58537E-05 | 0.000463984 | 7.779466566 | Eleostearic acid |
| 45 | 319.22769 | 13.685 | C20H32O3 | 0.007002216 | 0.038424658 | 6.594824089 | 11,12-Epoxy-(5Z,8Z,11Z)-icosatrienoic acid |
| 46 | 301.21713 | 14.429 | C20H30O2 | 3.7839E-06 | 0.000151012 | 5.345261627 | cis-5,8,11,14,17-Eicosapentaenoic acid |
| 47 | 329.24847 | 14.832 | C22H34O2 | 1.07022E-06 | 7.83045E-05 | 3.840511529 | all-cis-4,7,10,13,16-Docosapentaenoic acid |
| 48 | 281.24838 | 14.943 | C18H34O2 | 0.021523704 | 0.077450049 | 2.693684449 | Elaidic acid |
| 49 | 602.34656 | 14.727 | C28H50NO7P | 5.93595E-05 | 0.001042353 | 2.479698485 | LPC 20:4 |
| 50 | 500.27826 | 14.662 | C25H44NO7P | 2.03353E-05 | 0.000525129 | 2.280577218 | LPE 20:4 |
| 51 | 552.33087 | 14.607 | C24H48NO7P | 0.00033894 | 0.004021472 | 2.212629663 | LPC 16:1 |
| 52 | 798.56573 | 14.771 | C42H78NO7P | 7.2698E-06 | 0.00022796 | 2.02719572 | PC (16:2e/18:2) |
| 53 | 818.59229 | 15.663 | C42H82NO8P | 0.015288299 | 0.063316633 | 1.882069467 | PC (16:0/18:1) |
| 54 | 842.59229 | 16.589 | C44H82NO8P | 0.000917221 | 0.007804018 | 1.701416774 | PC (18:1/18:2) |
| 55 | 130.08708 | 2.517 | C6H13NO2 | 0.039323594 | 0.112830442 | 1.688196966 | 3-Amino-4-methylpentanoic acid |
| 56 | 124.00715 | 1.355 | C2H7NO3S | 0.008914294 | 0.046587801 | 1.439056183 | Taurine |
| 57 | 267.23282 | 14.825 | C17H32O2 | 0.005818056 | 0.033170476 | 1.432850036 | trans-10-Heptadecenoic Acid |
| 58 | 480.30969 | 15.002 | C23H48NO7P | 0.024643905 | 0.085862495 | 1.415265993 | LysoPE 18:0 |
| 59 | 331.26425 | 15.121 | C22H36O2 | 0.000212877 | 0.002748612 | 1.362076852 | Adrenic acid |
| 60 | 212.00229 | 6.573 | C8H7NO4S | 0.028624298 | 0.091058456 | 1.234599615 | 3-Indoxyl sulphate |
| 61 | 307.26422 | 15.225 | C20H36O2 | 0.036413669 | 0.106570672 | 1.206847202 | 11(Z),14(Z)-Eicosadienoic Acid |
| 62 | 164.07144 | 5.128 | C9H11NO2 | 0.026028222 | 0.087895304 | 1.20622148 | D-Phenylalanine |
| 63 | 776.58179 | 15.055 | C40H80NO7P | 0.000512598 | 0.005073407 | 1.203060485 | PC (14:0e/18:1) |
| 64 | 558.3573 | 15.023 | C28H52NO7P | 0.005356034 | 0.03177431 | 1.178286827 | LPC 20:3 |
| 65 | 241.21721 | 14.677 | C15H30O2 | 0.003887421 | 0.02556316 | 1.07720858 | Pentadecanoic acid |
| 66 | 608.39392 | 15.683 | C28H56NO7P | 3.09072E-05 | 0.000629072 | 1.056233108 | LPC 20:1 |
| 67 | 840.57635 | 15.864 | C44H80NO8P | 0.016317582 | 0.065151663 | 1.035026569 | PC (16:0/20:4) |
