# Supplementary material for: Multi-Omics Integration in Mice With Parkinson’s Disease and the Intervention Effect of Cyanidin-3-O-Glucoside
Source: Front Aging Neurosci. 2022 Apr 29;14:877078. doi: 10.3389/fnagi.2022.877078 (PMC9099026; doi:10.3389/fnagi.2022.877078)
Supplement: Supplementary file 2 [file Table_2.DOCX]

Supplementary Material

Table S2: The identification of the potential biomarkers of Cy-3-G-treated in PD

| No. | m/z | Rtmin | Formula | Pvalue | FDR | VIP | Name |
| --- | --- | --- | --- | --- | --- | --- | --- |
| Positive | | | | | | | |
| 1 | 303.23196 | 14.224 | C20H32O3 | 0.019124377 | 0.236962239 | 10.46123359 | (+/-)11(12)-EET |
| 2 | 282.27914 | 15.23 | C18H35NO | 0.018866809 | 0.236962239 | 10.36739984 | Oleamide |
| 3 | 104.10707 | 1.349 | C5H13NO | 1.20514E-05 | 0.003386445 | 5.699215937 | Choline |
| 4 | 808.58618 | 15.196 | C46H82NO8P | 0.030266398 | 0.282065756 | 5.129972474 | PC (18:0/20:5) |
| 5 | 169.03583 | 2.065 | C5H4N4O3 | 0.000489245 | 0.020732281 | 4.022355956 | Uric acid |
| 6 | 568.33978 | 14.566 | C30H50NO7P | 0.032044279 | 0.29362312 | 3.879557424 | LPC 22:6 |
| 7 | 301.21582 | 13.954 | C20H28O2 | 0.043892654 | 0.333346911 | 3.702655056 | Isotretinoin |
| 8 | 284.29495 | 15.622 | C18H37NO | 0.021453516 | 0.241137515 | 3.308497818 | Stearamide |
| 9 | 118.06533 | 7.064 | C8H7NO4S | 0.045863735 | 0.336201117 | 2.866589503 | Indole |
| 10 | 144.08101 | 7.063 | C10H9N | 0.020569614 | 0.236962239 | 2.501636241 | 6-Methylquinoline |
| 11 | 351.21652 | 13.384 | C20H32O6 | 4.20352E-06 | 0.001771782 | 2.249138204 | 11-Dehydro thromboxane B2 |
| 12 | 256.26355 | 15.108 | C16H33NO | 0.02944283 | 0.278879835 | 2.194908468 | Hexadecanamide |
| 13 | 153.12752 | 15.271 | C10H16O | 0.042606416 | 0.326520076 | 1.919194359 | D-(+)-Camphor |
| 14 | 136.07599 | 2.605 | C8H9NO | 0.004716973 | 0.09172323 | 1.889762785 | Acetanilide |
| 15 | 343.22607 | 14.139 | C20H32O3 | 0.002510332 | 0.06224147 | 1.615730112 | 16(R)-HETE |
| 16 | 354.26389 | 13.821 | C20H32O4 | 3.20507E-07 | 0.000270188 | 1.521079442 | 12-epi Leukotriene B4 |
| 17 | 206.0816 | 9.849 | C11H11NO3 | 0.045416097 | 0.335840082 | 1.498739781 | Cinnamoylglycine |
| 18 | 824.61731 | 15.725 | C47H86NO8P | 0.028855951 | 0.276426893 | 1.450038856 | PC (19:0/20:4) |
| 19 | 165.05496 | 1.569 | C9H11NO3 | 0.002137358 | 0.054599776 | 1.370480731 | 2-Hydroxyphenylalanine |
| 20 | 357.20349 | 13.299 | C20H30O4 | 0.00011505 | 0.010776382 | 1.216114247 | 13,14-dihydro-15-keto Prostaglandin A2 |
| 21 | 428.37427 | 13.803 | C25H50NO4 | 0.030448379 | 0.282065756 | 1.117635464 | ACar 18:0 |
| 22 | 375.21451 | 13.504 | C22H30O5 | 0.000160585 | 0.011281064 | 1.085435765 | 20-Dihydro 6-methylprednisone |
| 23 | 311.12418 | 6.221 | C19H20O5 | 0.000461209 | 0.020732281 | 1.068842118 | (4E)-1,7-bis(3,4-dihydroxyphenyl)hept-4-en-3-one |
| Negative | | | | | | | |
| 24 | 253.21721 | 14.568 | C16H32O3 | 0.012063467 | 0.135791331 | 11.15016062 | 16-Hydroxyhexadecanoic acid |
| 25 | 327.23291 | 14.635 | C22H32O2 | 0.002158374 | 0.07288663 | 9.17885947 | Docosahexaenoic acid |
| 26 | 303.23273 | 14.694 | C20H32O2 | 0.005870257 | 0.103715789 | 7.017552837 | Arachidonic acid |
| 27 | 319.22769 | 13.685 | C20H32O3 | 0.010866594 | 0.135791331 | 6.222650342 | 11,12-Epoxy-(5Z,8Z,11Z)-icosatrienoic acid |
| 28 | 301.21713 | 14.429 | C20H30O2 | 0.007991219 | 0.125290896 | 2.859514688 | cis-5,8,11,14,17-Eicosapentaenoic acid |
| 29 | 243.19644 | 13.998 | C14H28O3 | 0.03307873 | 0.279260813 | 2.831117539 | 2-Hydroxymyristic acid |
| 30 | 151.02589 | 2.082 | C5H4N4O2 | 0.02188046 | 0.228702902 | 2.814592894 | Xanthine |
| 31 | 329.24847 | 14.832 | C22H34O2 | 0.011467802 | 0.135791331 | 2.465158566 | all-cis-4,7,10,13,16-Docosapentaenoic acid |
| 32 | 180.06648 | 2.1 | C9H11NO3 | 0.030486539 | 0.267671814 | 1.581404853 | L-Tyrosine |
| 33 | 500.27826 | 14.662 | C25H44NO7P | 0.037398885 | 0.293766192 | 1.580050345 | LPE 20:4 |
| 34 | 602.34656 | 14.727 | C28H50NO7P | 0.037670054 | 0.293766192 | 1.494678005 | LPC 20:4 |
| 35 | 321.04379 | 9.595 | C10H15N2O8P | 0.040839636 | 0.301469191 | 1.155087862 | Thymidine 5'-monophosphate |
| 36 | 335.22287 | 13.214 | C20H32O4 | 0.000162998 | 0.02385207 | 1.101664682 | 15(S)-HpETE |
