# Supplementary material for: Multi-Omics Integration in Mice With Parkinson’s Disease and the Intervention Effect of Cyanidin-3-O-Glucoside
Source: Front Aging Neurosci. 2022 Apr 29;14:877078. doi: 10.3389/fnagi.2022.877078 (PMC9099026; doi:10.3389/fnagi.2022.877078)
Supplement: Supplementary file 3 [file Image_1.PDF]

Supplementary Material

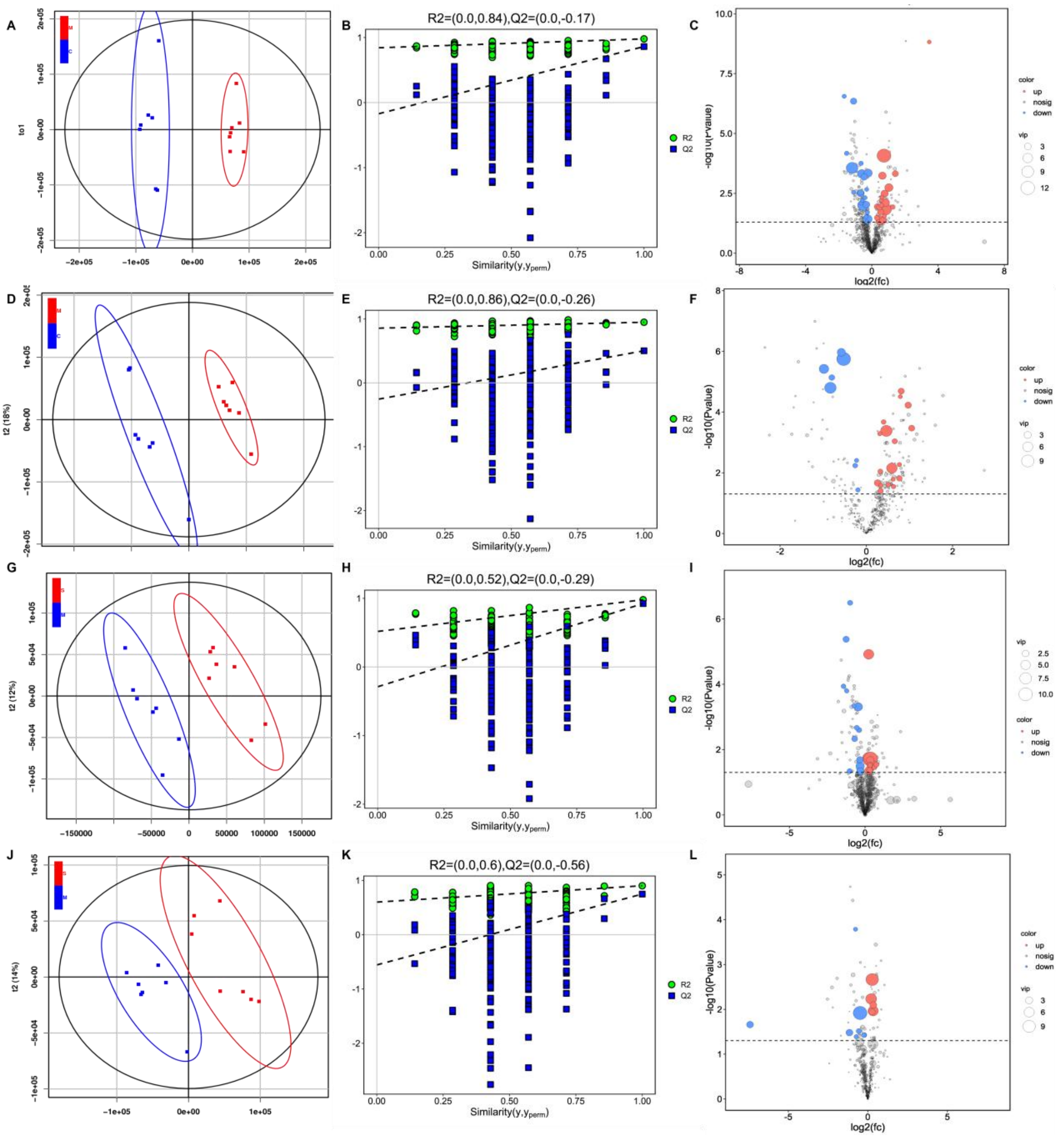

Figure S1: S-plot of the OPLS-DA model for the control and model group in positive ion mode(A) and negative ion mode (D); OPLS-DA loading plot for the control and model group in positive ion mode(G) and negative ion mode (J); Validation plot of the control and model group in positive ion mode (B) and negative ion mode (E) obtained from 200 permutation tests; Validation plot of the model and Cy-3-G group in positive ion mode (H) and negative ion mode (K) obtained from 200 permutation tests; Volcano plot between the control and model group in positive ion mode (C) and negative ion mode (F); Volcano plot between the model and Cy-3-G group in positive ion mode (I) and negative ion mode (L).
